# Modulating local airway immune responses to treat allergic asthma: lessons from experimental models and human studies

**DOI:** 10.1007/s00281-020-00782-4

**Published:** 2020-02-04

**Authors:** A.L. Voskamp, T. Groot Kormelink, R. Gerth van Wijk, P.S. Hiemstra, C. Taube, E.C. de Jong, Hermelijn H. Smits

**Affiliations:** 1 Department of Parasitology, Leiden University Medical Center, Albinusdreef 2 2333 ZA, Leiden, The Netherlands; 2 Department of Experimental Immunology, Amsterdam University Medical Centers, AMC, Amsterdam, The Netherlands; 3 Department of Internal Medicine, Section Allergology, Erasmus University Medical Center, Rotterdam, The Netherlands; 4 Department of Pulmonology, Leiden University Medical Center, Leiden, The Netherlands; 5 Department of Pulmonary Medicine, University Hospital Essen – Ruhrklinik, Essen, Germany

**Keywords:** Asthma, Allergic rhinitis, Dendritic cells, Th2 cells, Immune cells, Lung tissue, Nasal tissue, Human, Mouse

## Abstract

With asthma affecting over 300 million individuals world-wide and estimated to affect 400 million by 2025, developing effective, long-lasting therapeutics is essential. Allergic asthma, where Th2-type immunity plays a central role, represents 90% of child and 50% of adult asthma cases. Research based largely on animal models of allergic disease have led to the generation of a novel class of drugs, so-called biologicals, that target essential components of Th2-type inflammation. Although highly efficient in subclasses of patients, these biologicals and other existing medication only target the symptomatic stage of asthma and when therapy is ceased, a flare-up of the disease is often observed. Therefore, it is suggested to target earlier stages in the inflammatory cascade underlying allergic airway inflammation and to focus on changing and redirecting the initiation of type 2 inflammatory responses against allergens and certain viral agents. This focus on upstream aspects of innate immunity that drive development of Th2-type immunity is expected to have longer-lasting and disease-modifying effects, and may potentially lead to a cure for asthma. This review highlights the current understanding of the contribution of local innate immune elements in the development and maintenance of inflammatory airway responses and discusses available leads for successful targeting of those pathways for future therapeutics.

## Introduction

Asthma is a chronic inflammatory disease of the lungs resulting in episodes of reversible airway obstruction in a growing group of both children and adults [1]. Lung inflammation is a critical element in the pathogenesis of asthma. Interplay with local structural cells will lead to airway remodelling in response to various exogenous triggers, such as allergens, viral infections, air pollution, or cigarette smoke. Allergic asthma, in which Th2-type immunity plays a central role, represents the majority of asthma cases, particularly in children [2]. Advances in treating severe allergic asthma have been made through targeting specific components of adaptive immunity in the Th2-type cascade. On the horizon is the next generation of therapeutics with the aim of not only controlling symptoms but also addressing the underlying cause by targeting innate immunity and redirecting the adaptive immune response. Targeting innate processes and altering the immune response requires knowledge of cellular interactions and responses in the affected organ, i.e., the airways. This is relatively easily obtained from mouse models sensitized to allergens; however, their lung anatomy and immune system differs from that of humans, and they have a short lifespan, making it difficult to assess long-term effects of chronic inflammation [3]. On the other hand, disease development is difficult to assess in a human cohort, and the availability of airway tissue to study local responses is limited. Recent technical developments such as mass-cytometry and single-cell RNA sequencing may help to partly overcome this limitation [4, 5] by vastly increasing the volume of data that can be obtained from relatively small tissue or sputum samples. With these types of advances, our understanding of the mechanisms underlying allergic asthma is increasing and targets within the innate immune system are coming into view. In this review, we will discuss the therapeutic advances targeting innate immune components and highlight future high potential strategies.

## Biologicals targeting Th2-type immune responses: successful translation from bench to bedside

Evidence of the crucial importance of T(h)2 cell cytokines, eosinophils, and IgE in human asthma stems largely from experimental allergic airway models. These studies, combined with the presence of peripheral blood allergen-specific Th2 cells and eosinophils in allergic patients, have resulted in the generation of a new class of therapeutic antibodies against Th2-type components, so-called biologicals. These are recommended for severe asthma when conventional medication is not effective. Various biologicals targeting IgE, IL-4R alpha chain, IL-5, and IL-5R are approved (extensively reviewed elsewhere) [6], showing beneficial effects in particular subgroups of patients, but not in others. Clinical markers, including IgE levels, blood eosinophil count and exhaled nitric oxide (Fe_NO_) [7], are often a predictor of response and an indicator to which specific biological should be administered; however, a more complete profile is necessary to increase accuracy. For example, for omalizumab, the first approved biological targeting IgE, it was recently shown that a high baseline level of serum CXCL10 and IL-12 is predictive of a response to the treatment in severe asthma [8]. This not only predicts which patient groups benefit the most but also highlights the existence of different endotypes in allergy and asthma and the need for a more personalized approach in treating them. Another example of a novel approach is to block the prostaglandin D2 (PGD2) receptor (DP2 or CRTH2). DP2 is expressed on various Th2-related cell types, and when PGD2 is released by activated mast cells, type 2 cells will be recruited and activated and asthma development is accelerated [9, 10]. In clinical trials, DP2 antagonists have been well tolerated and shown potential efficacy through improvement of FEV_1_ and reduced airway eosinophils [11]. However, mostly patients with eosinophilic asthma seem to benefit from this therapy. When assessing efficacy, identifying the appropriate patient population can make the difference between success and discontinuation of the therapy.

Despite the advances in biologicals to treat asthma [12], so far, none have shown a long-lasting disease-modifying effect and termination of monoclonal antibody therapy usually results in a reoccurrence of symptoms. Indeed, stopping after even 5 years of omalizumab therapy resulted in an increase in exacerbations compared with patients who stayed on anti-IgE treatment [13], indicating that maintenance therapy is needed to achieve asthma control. In addition, current biologicals mostly target the end of the T(h)2 inflammatory cascade, affecting eosinophil activation and the IgE-mediated responses, while the process of allergic sensitization and early clinical symptoms remains untouched. It may be interesting, therefore, to address upstream targets in the allergic response to reach a more widespread suppression, leading to improved disease management.

With advances in cellular and molecular techniques, we are now gaining knowledge on essential elements of the inflammatory responses in the affected lung tissues of asthma patients. These studies confirm a crucial role of the barrier function of the epithelium, the cytokines they produce, dendritic cells (DCs) as orchestrators of the immune system, and a relatively new class of immune players, innate lymphoid cells. With this knowledge, new avenues of therapeutics should be pursued, targeting cells and molecules responsible for initiating and orchestrating the inflammation in asthma to achieve long-lasting modification of the immune response.

## Dendritic cells

Allergen-specific Th2 cells develop from naive T cells via stimulation by allergen-exposed dendritic cells (DCs) that migrated from peripheral sites, i.e., the lung, to the draining lymph nodes (LN). As such, DCs are the main cell type responsible for both the sensitization and induction of effector phases in allergies (reviewed in [14, 15] and are likely important targets for therapeutic interventions to control allergic airway disease. To identify DC-specific therapeutic targets, detailed knowledge on the presence and functions of distinct DC subsets in the airways, and in allergic airway disease, is crucial.

In both humans and mice, DCs are classified as conventional DCs (cDCs), consisting of a cDC1 and cDC2 subset, and plasmacytoid DCs (pDCs). It was only recently that murine and human DC populations were aligned across various tissues including the lung, enabling better comparison of DC subsets between species. In brief, expression of CD11c and MHCII together with additional markers define cDCs subsets: where cDC1 express CADM1, XCR1, and IRF8; cDC2 express CD172a, CD1c, and IRF4; pDCs express MHCII and CD123, but not CD11c, with high IRF8 and intermediate IRF4 expression [16]. However, in many studies published so far, human and mouse DC subsets have been classified by other separate, non-species overlapping markers: human cDC1s and cDC2s have been referred to as CD141^+^ and CD1c^+^ DCs, while in mice, cDC1 have been identified as CD8a^+^CD11c^hi^ or CD103^+^, and cDC2 as CD8a^−^CD11c^hi^ or CD11b^+^, respectively. Moreover, the presence of monocyte-derived inflammatory DCs (moDCs) has been described in multiple tissues and is often characterized by the additional expression of CD11b, CD14, CD64, and/or the high-affinity Fc receptor for IgE, FcεRI [17–19]. Importantly, due to limited use of cell-identification markers in many studies, these moDCs may also be present in the gated cDC2 population, which hampers the interpretation, comparison, and translation of results from various studies in human and murine (model) systems.

Insights into cell distribution in the airways during allergic disease can be important as distinct DC subsets have been shown to exhibit differential functional capacities that are largely conserved between species (reviewed in [14, 20]. Generally, cDCs are specialized in antigen-specific stimulation of T cells. cDC1 can induce CD4^+^ Th1 cell responses and have cross-presenting capacity enabling them to activate CD8^+^ T cells by presentation of extracellular-derived antigens in MHCI. cDC2 have a more prominent role in the induction of either effector Th cell or Treg cell responses, depending on activating or tolerizing signals they receive during their time as sentinels in the peripheral tissue. In contrast, pDCs are potent IFNα producers and are primarily involved in anti-viral immune responses, though tolerance-inducing capacities have also been reported. It is unclear what causes the functional divergence of DC subsets, though it is likely due to a dynamic interplay of intrinsic differences (e.g., in antigen uptake, lysosomal processing, migration) and context-dependent conditions (e.g., type of antigen, adjuvant, dose, and tissue environment) [21]. These context-dependent conditions also significantly contribute to the divergence in T cell–polarizing capacities of single DC subsets in the airways, as discussed below.

### The role of various DC subsets in asthma model systems

The individual contribution of the distinct DC subsets in the sensitization and effector phases of allergic airway disease has been almost exclusively studied in murine asthma models. In general, airway challenges result in DC migration to the mediastinal lymph nodes (MLN) after 1 day, with highest frequencies of cDC2s, followed by cDC1 and pDCs. Monocyte-derived DCs are poorly migratory [17, 19, 22], which probably excludes any possible contribution to sensitization. However, no consensus has been reached on which subset is dominant in orchestrating allergic airway disease.

Of all the DC subsets, cDC2s take up allergens most efficiently, migrate to the draining LNs, and induce T cell proliferation [17, 23]. Two studies demonstrated that cDC2s were able to induce Th2 and Th17 cell–mediated asthma in vivo, or upon adoptive transfer of house dust mite (HDM)-primed and sorted lung DC subsets into naive recipients [17, 24]. In contrast, Nakano et al. demonstrated that isolated lung cDC2s from ovalbumin-, HDM-, or cockroach-immunized mice were critical for enhanced Th1 cell responses [22]. However, using a different marker to evaluate the role of cDC2, i.e., the transcription factor IRF4, it was shown that mice, either deficient in or depleted of, IRF4 had reduced Th2 cell responses in the lungs and skin [25, 26]. Although there is some conflicting evidence, overall cDC2 seemed potent in their ability to drive allergen-specific Th2 cell responses in the lung.

The role of cDC1s is well appreciated in the induction of anti-viral and anti-tumor immunity; however, their role in allergen-specific Th2 cell polarization remains controversial. cDC1 only poorly take up allergens compared to other DC subsets [17, 22] and opposing studies have indicated that they either promote or inhibit Th2 cell immune responses in the lung [27–29]. This may be related to the type and amount of allergen used. This subset may also have a tolerogenic function, as cDC1s from tolerized mice induced Foxp3 regulatory T cells (Treg) cells in vitro, and tolerization to inhaled antigen was impossible in cDC1-deficient mice [30].

Studies on moDC function in the lungs during allergen challenge show potent allergen presentation and abundant release of proinflammatory chemokines (especially because of their high prevalence) that influences eosinophil and monocyte migration a few days after repeated allergen challenge [17, 31] and attracts Th2 cells [17, 32]. The findings point to a crucial role in promoting existing allergic inflammation in the lung, while the role of moDCs in the sensitization phase was limited due to their poor migratory capacity and need for high HDM doses to induce allergic asthma sensitization [17].

Finally, a critical role was suggested for pDCs in mediating allergic airway disease during respiratory viral infections, including rhinovirus-induced exacerbations [33, 34]. Enhanced Th2 cell responses were induced by IL-25-activated pDCs that were recruited to the lung within 1 day after virus-induced exacerbations [33]. This is supported by observations in humans, showing that IgE-activated human pDC drive enhanced Th2 cell polarization [35]. In contrast, in neonatal mice, pDC-derived semaphorin 4a induced the expansion of Treg cells, which controlled susceptibility to viral bronchiolitis and subsequent viral challenge–induced asthma in later life [34]. The discrepancies found in pDC functions may be at least partly related to the timing of the analyses: e.g., before or after initiation of inflammation, as immature pDCs more likely enhance tolerance.

Collectively, even though the cDC2 subset seems most capable of driving allergic inflammation in the lung, it is clear that the other DC subsets can also gain the capacity to drive Th2 cell responses, depending on the model, the type of allergen, or allergen dose. Until now, functional analysis of human lung DC subsets in healthy individuals or asthma patients is lacking, and it remains difficult to extrapolate the findings from mouse models. Yet, the mechanisms employed by distinct DC subsets to enhance Th2 cell–mediated inflammation generally appear to be more alike and will be discussed below.

### Mechanisms involved in Th2 cell induction by DCs

The mechanisms through which DCs control Th2 cell polarization appear to vary both in murine disease models as well as between humans, which can likely be attributed to species differences, as well as differences in allergen properties, microenvironmental conditions, and genetic variations in the host. Importantly, DCs do not produce IL-4, the key driver of Th2 cell polarization, which may instead be produced by local accessory cells such as basophils or innate lymphoid cells (ILCs) [21]. OX40L, expressed by DCs, along with the notch ligands Jagged1/2, is well recognized for their ability to effectively induce and/or enhance Th2 cell differentiation, in part by regulating the expression of IL-4 and the Th2-specific transcription factor GATA3 in T cells [36–38]. In addition, suppression of IL-12 production by DCs is a pre-requisite to enable induction of Th2 cell responses in mice and humans because of the absence of Th2 cell–suppressive, counteracting Th1 responses [39, 40]. A variety of additional receptors expressed on DCs have been associated with Th2 cell differentiation, including the costimulatory molecules PD-L2 [41], ICOSL [42, 43], CD40 [44], and the pattern recognition receptors Dectin-1 and Dectin-2 [45–47], DC-SIGN [48] and mannose receptor (MR) [49], and the high affinity Fc receptor for IgE, FcεRI [50]. However, most of these receptors have also been implicated in the induction of other T cell effector phenotypes [50, 51], and moreover, regulation of Th2 cell development via these receptors often follows similar mechanisms, i.e., modulation of IL-12 release and OX40L or Jagged1/2 expression by DCs.

Importantly, studies on monocytes and DC subsets from allergic and non-allergic subjects also point to an important role for OX40L and IL-12 in allergy and asthma. Multiple studies have looked at activated moDCs and cDC2s derived from allergic rhinitis, allergic asthma, and/or atopic dermatitis patients compared with non-allergic controls, describing reduced IL-12 release accompanied by increased release of pro-allergic factors, (such as PGE2), proinflammatory cytokines (TNF-α, IL-1β), and type 2 chemokines. Furthermore, cDC2s from those patients also had increased expression of costimulatory molecules OX40L, PD-L2, and cytokine receptor TSLPR, leading to enhanced Th2 and Th17 cell differentiation. In addition, their pDCs produced less IL-12, and IFNα, resulting in a reduced capacity to induce IL-10-producing CD4^+^ T cells [41, 52–54]. Lastly, FcεRI expression and IgE binding on pDCs and cDC2s is significantly higher in allergic patients than in healthy individuals [50, 54, 55] and IgE-mediated activation of cDC2s is mostly associated with induction of Th2 cell responses [56]. Collectively, these findings indicate that functional anomalies in DC subsets of allergic patients, either intrinsic or induced by allergic inflammation, lead to changes in costimulatory molecule expression and cytokine/chemokine release, together contributing to enhanced Th2 cell development. OX40L and IL-12 appear to be most consistently associated with increased Th2 cell development, suggesting that targeting OX40L and/or enhancing IL-12 secretion may attenuate Th2 cell polarization, and consequently Th2 cell–mediated airway diseases.

Administration of IL-12 has been tested in a group of mild asthmatic patients and resulted in decreased numbers of circulating eosinophils after allergen challenge; however, no change was observed in sputum eosinophils, late-phase response, or airway hyperresponsiveness. Additionally, > 20% of patients suffered from flu-like symptoms, abnormal liver functions, or cardiac arrhythmias [57], precluding this as a viable treatment option. Blocking OX40L-mediated signaling has also been tested in human clinical trials. A phase II trial of a humanized IgG1 anti-OX40L monoclonal antibody (Oxelumab), in allergic asthma patients, revealed pharmacological activity through decreased total serum IgE and airway eosinophils after 16 weeks of treatment. However, there was no effect on the primary outcome, allergen-induced airway responses, possibly due to insufficient dosing and treatment duration or an inadequate outcome parameter [58]. Oxelumab was discontinued following this phase II trial; however, KyMab produced an alternative IgG4 anti-OX40L Mab (KY1005) which was able to block T cell–driven skin inflammation while being well tolerated in phase I clinical trials in healthy volunteers [59]. KyMab are currently conducting a phase IIa clinical trial for the treatment of atopic dermatitis, with preliminary results expected in the first half of 2020 [59, 60]. These results will give an indication of the efficacy of KY1005 in treating Th2-type related diseases, and whether this is applicable to asthma.

It should be noted, however, that OX40L is not only associated with enhanced Th2 cell development. Others have also described essential contributions of OX40L in Treg, Th1, and Tfh cell development in both mice [61, 62] and humans [61, 63]. This implies that OX40L-mediated Th2 cell development is, at least partly, dependent on additional signals (like cytokines) and that inhibition of OX40L signaling may not necessarily result in attenuation of Th2 cell–driven inflammation only. Therefore, it may be more fruitful to target pathways that lead to modification of dendritic cell function, rather than targeting single costimulatory molecules or DC cytokines.

## Epithelium-derived innate cytokines driving DC activation and innate lymphoid cells

Biologicals targeting IL-13, IL-13R, IL-5, or IL5R will neutralize type 2 cytokines. This, however, does not prevent the activation of Th2 cells, and alternative approaches targeting upstream processes in the Th2-type cascade have been suggested. A major producer of these cytokines, in addition to Th2 cells, is type 2 innate lymphoid cells (ILC2). These cells are defined as primarily tissue-resident lymphocytes, which lack antigen-specific B or T cell receptors. ILCs rapidly produce various cytokines in response to viral, microbial, or parasitic encounter, or tissue damage [64]. Three subclasses of ILCs have been identified in parallel to the different effector Th cell subsets, based on their cytokine profile and transcription factor expression, with ILC2s expressing GATA-3 and producing IL4, IL5, and IL-13 upon activation [65]. In fact, depending on the allergen or route of exposure in mouse models of allergy, activated ILC2 provide an early type 2 cytokine response, which stimulates Th2 cell skewing [66]. However, it is unclear whether ILC2s also provide an early source of type 2 cytokines in humans or whether their role is more prominent in ongoing inflammation. Murine and human ILCs are functionally and phenotypically similar, although their phenotype can differ depending on the tissue in which they reside. A recent study utilizing mass-cytometry to identify ILC subtypes within various human tissues concluded that ILC2s and ILC3s were under-represented in non-mucosal tissue and the lung, where the majority of innate lymphoid cells were NK cells. This is in contrast to the lungs in mice, where the majority of ILCs are represented by ILC2s [67]. In agreement with murine studies of allergic airway inflammation, however, increased numbers of ILC2s are present in the blood and airways (as determined in BAL, sputum, or sinonasal mucosa) of asthmatic patients, in particular those with uncontrolled or partially controlled asthma [66, 68, 69]. Additionally, rapid recruitment of ILC2s upon allergen exposure has been observed.

### TSLP, IL-33, and IL-25

Accumulating evidence suggests that the epithelial barrier integrity at the antigen contact site will influence the subsequent immune responses by facilitating penetration of allergens into the submucosa [15]. Various studies have shown that epithelial exposure to environmental insults, such as allergens (in part through proteolytic activity [70]), virus infection [71], or air pollutants [72], may serve as a trigger for the epithelial release of the innate type 2 skewing cytokines TLSP, IL-33, and IL-25, often referred to as alarmins [15, 73]. These cytokines bind to and activate many different cell types; however, in the context of the initiation and perpetuation of allergic responses in the airways, both DCs and ILC2 are important players. In both murine and human DCs, one or more of the following Th2 cell inducing characteristics are initiated by each of these cytokines [73, 74]: (1) DC maturation (enhanced MHCII and costimulatory molecule expression) but without the induction of IL-12 secretion, (2) OX40L expression, and (3) secretion of Th2 cell–attracting chemokines (like CCL17 and CCL22) [52, 75, 76]. In human ILC2s, stimulation with TSLP has been shown to promote cell survival, whereas IL-33 enhances cell activation and type 2 cytokine release [77, 78]. Furthermore, in mouse models of allergic airway inflammation induced by IL-33, steroid treatment affects Th2 cells but not ILC2s, and this resistance is mediated by TSLP [79]. Indeed, higher levels of ILC2s have been detected in the lungs, sputum, and blood of steroid-resistant compared with steroid-sensitive asthma patients [68]. Moreover, the lung, but not blood ILC2s, from asthmatic patients with elevated TSLP levels was found to be steroid resistant. This could be reversed by inhibitors of MEK and STAT5, components of the TSLP signaling pathway [80]. In contrast, a recent study in children with severe steroid-resistant asthma shows that airway ILC2 may be sensitive to steroid treatment, as shown using cultured cells as well as by intramuscular administration of a systemic steroid. This treatment was found to reduce exacerbations and symptoms as well as reducing ILC2 and Th2 cells in induced sputum, without affecting IL-17^+^ ILC or Th17 cells [81]. Therefore, steroid resistance of ILC2 may differ between children and adults. Importantly, anti-TSLP (tezepelumab) has reached phase IIb clinical trials in adults, showing a significant reduction in the annual asthma exacerbation rate compared with placebo in patients with severe, uncontrolled asthma [82]. Whether such treatment restores steroid-sensitivity of ILC2 remains to be determined.

IL-33 had previously been shown to remain elevated despite maximal steroid treatment in pediatric severe therapy-resistant asthma [83]. Furthermore, murine studies showed that blocking of IL-33 by targeting the IL-33 receptor ST2, by anti-IL-33 or by synthetic immunomodulatory peptides, was very effective in reducing OVA-induced airway inflammation, more so, in fact, than blocking individual Th2-type cytokines IL-4 or IL-13 [84–86]. Currently, anti-IL33 receptor (ST2) antibodies are in early phase clinical trials to assess their safety and efficacy in subjects with moderately severe asthma [59]. Direct targeting of IL-33 has also been considered in humans and so far a humanized anti-IL-33 was found to be safe in healthy subjects in a phase I clinical trial [87].

The development of an anti-IL-25 biological has been slower than that of IL-33 or TSLP, in part due to the difficulty in generating this antibody. Nevertheless, an anti-IL25 antibody is now in pre-clinical development and has been shown to suppress RV infection induced airway inflammation, while improving anti-viral responses in a mouse model of OVA-induced allergic airway disease [88]. Interestingly, within the airway epithelium, a relatively rare population of chemosensory cells (also called Tuft or brush cells) likely serves as the most important cellular source of IL-25 [89].

Even though existing approved biologicals against IL-5 or IL-13 (receptors) will also be able to neutralize the activity of those cytokines being produced by ILC2 and not only TH2 cells, it is questionable whether these biologicals can reach these targets locally, as ILC2 primarily resides in the (lung) tissue. Anticalins, a new class of biopharmaceuticals, may overcome this issue. Anticalins are lipocalin molecules that can be engineered to target proteins of interest but are smaller than antibodies and have better tissue penetration [90]. An IL4-Ra targeted anticalin delivered through oral inhalation is currently in phase I clinical trials [59]. Further studies are needed to investigate the efficacy of this highly tissue-penetrating class of drugs, and whether it will be more effective in asthma patients.

Granting that the above described novel therapeutics are targeting molecules more upstream of the allergic cascade, the efficacy of these therapies still relies on blocking effector molecules rather than changing the function of cells that play a crucial role in initiating and propagating local allergic airway responses. Therefore, it will be important to further explore avenues more focused on modulating immune cell function, with the aim of changing its activity rather than temporarily blocking it.

## Immunostimulatory adjuvants for immunotherapy

In contrast to therapies with biologicals that only seem to dampen certain aspects of allergic inflammation, allergen-specific immunotherapy (AIT) is the only treatment available, which can cure and prevent allergic symptoms. AIT has been shown to be effective in allergic rhinitis and in venom allergies [91] and to a lesser extent in allergic asthma; however, the treatment duration is between 3 and 5 years, and a large number of administrations are required to reach efficacy. Successful AIT is associated with a variety of changes at the cellular level, such as a shift from Th2 to Th1 cell responses and the induction of tolerogenic responses. The desired immune responses during AIT can be modulated and improved by immunostimulatory/regulatory adjuvants acting on DCs, leading to an earlier and longer-lasting effect. For example, the use of TLR ligands, vitamin D3, and probiotics has been proposed.

TLR ligand adjuvants are bacterial derived compounds, which can be combined with the allergen to induce a Th1 type immune response, thereby attenuating Th2-type responses. An example is Pollinex Quattro (PQ), which combines pollen allergens with the TLR4 ligand monophosphoryl lipid A (MPLA), the non-toxic variant of lipopolysaccharide. Several phase III clinical studies provide evidence that this product is well tolerated, with clinical efficacy and potent T cell responses [92]. The product is available (primarily) in Europe on a named-patient basis; however, the most recent phase III study of PQ Birch did not show a statistically significant difference between the active and placebo arms for the primary endpoint of combined symptom medication score averaged over the peak birch pollen season. This outcome affects the progress towards full registration and entering the US market. Results of a similar phase III study for PQ Grass due in the next year will determine whether full registration of PQ AIT will be pursued further [93].

TLR9 ligand CpG has also been tested as an adjuvant for AIT [94]. Although the primary endpoint of vascular permeability of nasal epithelium was not reached, patients treated with a ragweed allergen linked to CpG in a phase II study had reduced peak-season rhinitis symptom scores during both the first and second ragweed pollen seasons following treatment, and reduced allergen-specific IgE levels [94]. However, following another phase II/III trial in which clinical improvement did not reach significance in ragweed allergic patients [95], this particular therapeutic was discontinued. A similar approach was taken for HDM allergy, whereby the allergen was co-encapsulated with CpG in virus-like particles, showing reduced symptoms and increased allergen-specific IgG in a phase I/IIa study [96]. Additional phase II trials were then conducted with these particles but *without* the allergen. In these studies, symptom and medication scores improved [97], and asthma control was maintained during steroid reduction in allergic asthma patients, suggesting that a general modified immune function of DCs would be sufficient to change the development of allergen-specific T cell responses [98]. Although these types of adjuvant have shown efficacy in multiple clinical trials, it should be noted that efficacy is measured in comparison with placebo and not standard AIT without the adjuvant, making it difficult to assess its added value.

Oral application of bacterial lysates has been used to prevent respiratory tract infections for decades in middle-European countries. OM-85 is used most often, which is an extract of respiratory pathogenic bacteria [99]. Following the oral route, they modulate immune responses in the intestines, leading to increased immune maturation and immunity against respiratory pathogens [100]. Recent studies suggest that bacterial lysates also reduce virus-induced wheezing episodes with 30% in pre-school children with recurrent wheezing [101, 102]. In older children with asthma, bacterial lysates form an add-on therapy preventing disease exacerbations [103]. It is unclear how long-lasting the effect is and whether this spans over several seasons or years. Currently, the application of bacterial lysates is being studied to prevent recurrent wheezing and asthma in young infants [104].

Other adjuvants with immunoregulatory properties, as opposed to immunostimulatory properties, have also been considered. The risk of developing allergies has been correlated with low vitamin D levels [105]. Indeed, the active form of vitamin D, 1,25dihydroxy vitamin D3 (Vitamin D3), has immunomodulatory properties. Vitamin D3 modulates the function of a wide range of immune cells, including DCs, macrophages, T lymphocytes, and B lymphocytes, resulting in a regulatory response. In DCs that express the Vitamin D receptor (VDR) constitutively, Vitamin D3 prevents the full maturation of the cell, as well as the production of proinflammatory cytokines, in favor of tolerance-associated molecules such as ILT3 and IL-10. Furthermore, Vitamin D3 can repress OX40L expression by DCs [106]. Due to these effects, Vitamin D3–primed DCs induce regulatory T cells. Indeed, injection of Vitamin D3 in a human explant model induces dermal DCs with tolerogenic properties [105]. Furthermore, application of Vitamin D3 together with AIT significantly potentiates the beneficial in vivo tolerogenic responses in mouse models for allergic asthma, such as reduced airway hyperreactivity, airway eosinophilia, serum IgE, and Th2 cell responses, together with increased Treg cells and IL-10 in the lungs [107, 108]. In a placebo-controlled, randomized trial with allergic rhinitis patients, it was found that Vitamin D3 alleviates symptoms of allergic rhinitis, in both adults and in children [109, 110].

Despite promising pre-clinical studies, the realization into clinical efficacy can be difficult to achieve. The heterogenicity of humans and the broad range of disease endotypes involved in asthma are contributing factors to this, but in addition, the primary outcome chosen may not always represent the true efficacy of the drug. In many cases, subjective endpoints are assessed, which may be more susceptible to the placebo effect [111]. As discussed earlier, various DC subsets are involved in antigen recognition and the initiation of an immune response. Although many of the adjuvants discussed can induce a particular Th response, no specific DC subset is currently targeted directly, which may substantially improve the induction of more tolerogenic responses and down-modulation of pro-allergic Th2-type responses.

### Microbiome and “old friends”

It has been hypothesized that the rise in inflammatory diseases such as asthma, in westernized areas in the past 50 years, is the result of lifestyle changes and a reduced microbial exposure. This may result in insufficient priming and education of the neonatal immune system and subsequently, an increased risk of inflammatory diseases (Hygiene Hypothesis [112]). One of the earliest and most substantial microbial stimuli neonates encounter is by the microbiome. A diminished diversity of the microbiome composition, as a consequence antibiotic use in the first year of life and lifestyle changes, is linked to an increased risk of allergic diseases, such as asthma.

Multiple studies in both mice and humans have shown that absence of specific strains in gut microbiota were linked to increased (risk of) asthma development [113]. Furthermore, distinct unfavorable profiles of lung microbiota are related to specific endotypes of asthma [114–116]. Although supplementation with one of these specific strains has not shown strong evidence of preventing asthma [117], several components derived from the microbiome, such as Sema4a, D-tryptophan and short chain fatty acids, are being investigated for their immunoregulatory effects and have shown positive results in murine studies [34, 118–120]. Furthermore, probiotics have been combined with peanut oral immunotherapy showing sustained unresponsiveness (up to 4 years) to the allergen in the treated group compared with placebo [121, 122]. Again, further studies are required to assess its effectiveness over standard OIT.

Graham Rook has refined the hygiene hypothesis by introducing the term “old friends” to emphasize the crucial role of certain micro- and macrobionts that the human species has co-evolved with, while other (inhalant) pathogens or childhood infections do not seem to be linked to this protective effect [123]. These inhabiting “guests” can impose optimal immune shaping, in particular on the innate and regulatory arm of the immune system [123]. Examples of those “old friends” are helminth parasites, hepatitis A virus, toxoplasma, and *Helicobacter pylori*, a bacterium infecting the stomach, all showing protective associations in epidemiological studies with protection against the development of asthma [123–125]. Different model systems have confirmed this and revealed underlying immune mechanisms involving tolerogenic functions of various immune cells, including DCs, M2 macrophages, or regulatory T and B cells, which then suppress the development of Th2 cell responses and allergic inflammation [126]. Furthermore, helminth parasites, such as *Heligmosomoides polygyrus*, also suppress and neutralize allergic inflammation driven by innate cytokines produced by bronchial epithelium [127]. In population studies, it was already suggested that infections at an early age have a more dominant impact. Indeed, infection with *H. pylori* has been shown to be protective in neonatal mice and to a lesser extent in adults. The protective effect can even be transmitted transmaternally: both for helminths, like schistosomes, and for *H. pylori* [128, 129]. This effect was linked to skewing of regulatory T cells over effector T cells and (de)methylation of the Th2 cytokine genes versus the forkhead box P3 (FOXP3) locus [128, 129].

Interestingly, live infections are not necessary to suppress the development of allergic airway disease. Application of a lysate of *H. pylori* bacteria or secretory/excretory products of *H. polygyrus* or *Schistosoma mansoni* eggs was equally effective in suppressing different features of allergic airway disease [130–132]. For *H. pylori* lysate, this was also the case in different therapeutic settings, making it more interesting to investigate the potential application of a microbial derived molecule for the treatment of asthma [133]. From both helminth parasites as well as *H. pylori*, various molecules have been identified that can mimic the protective activity of a full infection [134, 135]. For example, *H. polygyrus* HpARI [136], ES-62 and AvCystatin from *Acanthocheilonema viteae* [134, 135], AIP-2 from hookworms [137], and *H. pylori*–derived vaculating cytotoxin A (VacA) [138] and gamma glutamyl transferase (ggt) [139] have also shown to suppress allergic inflammation in different models [134]. In addition, other molecules with a defined effect on the immune system have been described, driving either Treg or Breg responses and modifying DC function, such as *H. polygyrus* TGM [140], *S. mansoni* omega-1 [49, 141] or IPSE [142]. Further research is needed to assess these molecules as potential therapeutics for the treatment of allergies and asthma in humans, and whether these molecules can be incorporated in running AIT protocols.

## Early life immunity

In the search for novel therapeutics to treat or prevent the development of allergic airway diseases, it is important to consider the age of the target population. Allergy prevention would require modulation of immune responses in early life. Studies have shown that early life immunity differs from that of adults, which must be taken into consideration in the development of novel prevention and treatment approaches. These differences are partly due to maternal imprinting of the fetal immunity during its stay in the uterus, preventing detrimental maternal immune responses, and partly because the neonatal immune system is still immature and has not yet reached its full potential.

Multiple studies have shown that there is a Th2/Treg bias in early life. Several factors that contribute to this bias have been identified in mice. Neonatal T cells produce IL-4 more readily upon TCR stimulation than adult T cells, due to hypomethylation of the Th2 cytokine regulatory region [143]. Furthermore, neonatal, but not adult, Th1 T cells express both IL-13Ra1 and IL-4Ra, and undergo apoptosis when exposed to IL-4 [144]. Neonatal DCs contribute to the early Th2 bias through insufficient production of IL-12 compared with adult DCs. Maturation of these DCs at day 6 after birth overcomes this Th2 bias through increased production of IL-12 [145]. Enhanced Th2 responses in the neonate lung are also linked to a hyperactive IL-33 axis in early life. Epithelial IL-33 is unregulated after birth, resulting in an increase in ILC2s, eosinophils, basophils, and mast cells during remodelling in the developing lung. Exposure to allergen in this period results in a further increase of IL-33 production and induction of a Th2 type response to allergens [146, 147]. Other factors maintaining a Th2 bias after early life may also play a role. During the first 2 weeks after birth, the lung is gradually colonized by microbiota, which is associated with decreased allergen responsiveness and the emergence of Helios negative Treg cells, dependent on PD-L1 for development. Dysregulation of the formation of lung microbiota can therefore contribute to sustained Th2 bias and increased risk of allergic airway inflammation in adulthood [148]. Indeed, in humans, gut- and respiratory microbiota patterns at 2 months are associated with recurrent respiratory tract infections in the first year and later asthma development [149].

As a model for human neonatal immunity, cord blood cells are often used. These studies have revealed decreased monocyte and DC production of Th1-skewing cytokines IL-12 and type 1 interferons at birth compared with adults. Conversely, these cells produce as much or more IL-1β, IL-6, IL-23, and IL-10 compared with adult cells, supporting Th17- and Th2-type immunity [150, 151]. Differences between allergic and non-allergic infants in the further development of their immune system have also been observed. Non-allergic infants show progressive and significant age-related increases in TLR-induced innate cytokine production (IL-1β, IL-6, TNF-α, and IL-10) from birth to 5 years of age, with a parallel increase in adaptive Th1 cell responses. Allergic infants, in contrast, showed a relative decrease in these responses [152]. Although cord blood samples provide the easiest access to neonatal immune cells, a recent study using mass-cytometry showed that cord blood measurements are not predictive of postnatal immunity. In total, 15 of 21 immune cells, including cDC and pDC, measured in cord blood did not correlate with those measured in peripheral blood at 1 week after birth. Furthermore, cord blood values also differed from peripheral blood values taken at birth, implying that there are tissue differences between cord and peripheral blood as well as continuous changes over time [153]. In fact, the data revealed marked changes in immune components from birth to 3 months and followed a stereotypic pattern for all 100 children within the study, which was not predictable from cord blood measurements. The immunological changes detected were linked to interaction with microbes and found to be hampered in children with gut bacterial dysbiosis. Ultimately, these types of studies, with careful consideration of the source of cells, should help to identify immune gaps and (microbial) adjuvants as targets to set-up preventative DC-based therapies in children at risk for allergic disease.

## Concluding remarks

**Fig. 1:**
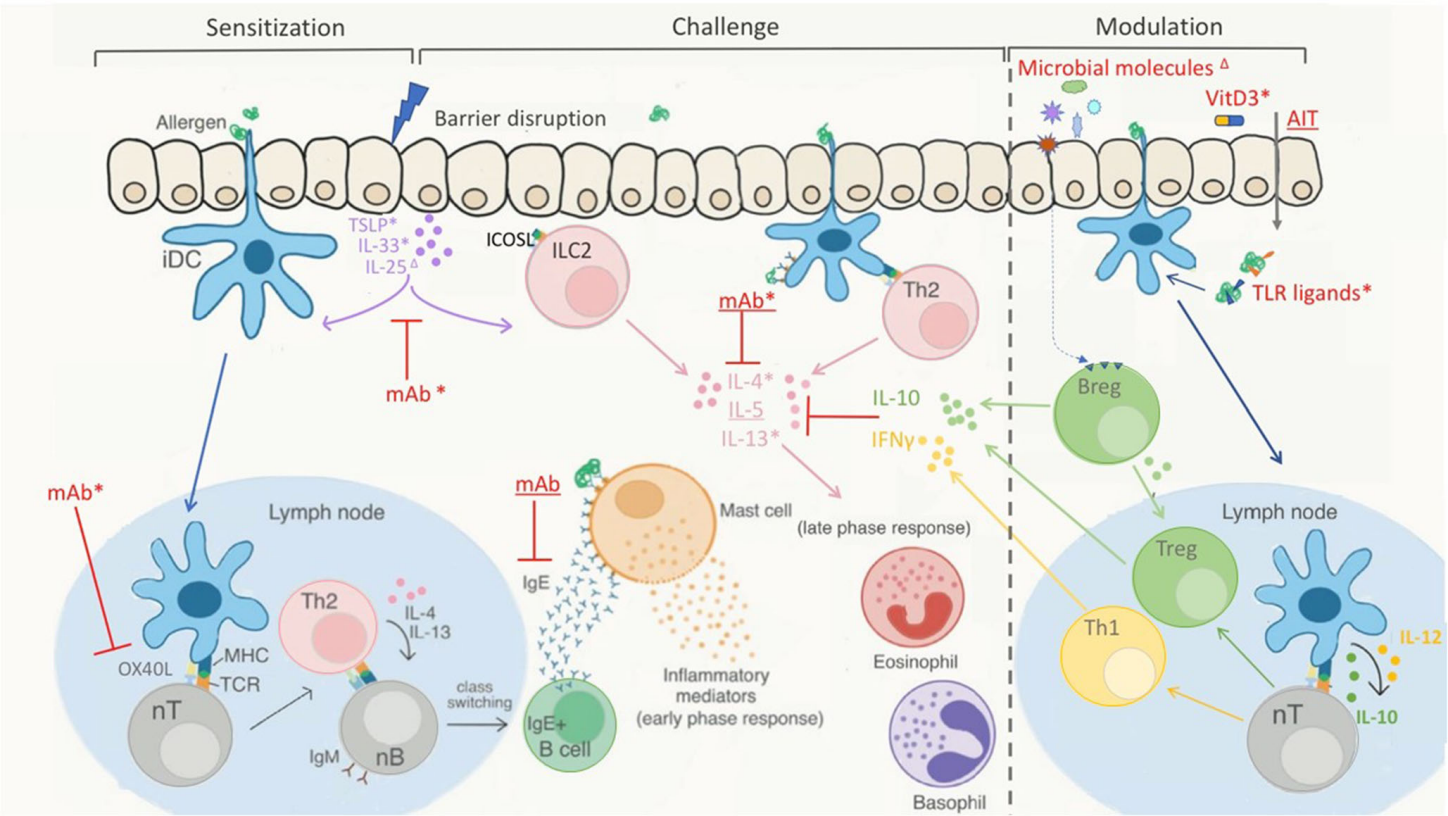
**Allergen-induced Th2-type response and targets for intervention/modulation.** During sensitization, immature dendritic cells (iDC) encounter allergens at the epithelial barrier of mucosal tissue. Upon allergen uptake, DC mature and migrate to the lymph node to induce differential and clonal expansion of allergen-specific Th2 cells from naive CD4^+^ T cells (nT). Th2 cell polarization can be facilitated by alarmins (TSLP, IL-33, IL-25) produced by disrupted epithelial cells, which induce OX40L upregulation on DC and activate ILC2s to produce Th2-type cytokines. In the lymph node, Th2-primed T cells produce IL-4 and IL-13 which initiates immunoglobulin class switching in allergen-specific naive B cells (nB), resulting in allergen-specific IgE producing plasma cells and IgE^+^ memory B cells. Upon subsequent allergen encounter (challenge), mast cells and basophils are activated through cross-linking of FceRI by allergen-specific IgE, producing inflammatory mediators responsible for the early phase allergic response. A late-phase response is initiated upon infiltration of additional effector cells to the site of allergen encounter. The Th2-type response to the allergen is further maintained and reinforced by stimulated allergen-specific Th2 cells. Interventions mediated by biologicals (monoclonal antibodies; mAb) and therapies designed to modulate the immune response (on the right) are indicated in red. Intervention is achieved through blocking of IgE, Th2 cytokines (IL-4, IL-5, IL-13), and/or their receptors. Intervention earlier in the Th2-type cascade could be achieved by blocking alarmins or costimulatory receptors such as OX40L and ICOSL. Modulation involves redirection from a Th2- to a regulatory- and/or Th1-type immune response. Interventions/modulatory therapies in pre-clinical stage are indicated with Δ, those in clinical trials are indicated with *, and those already registered for use are underlined

Fundamental research in pre-clinical models of allergic asthma has paved the way for the development of biologicals targeting key Th2-type responses (Fig. 1). These drugs form a break-through as they offer a solution for specific endotypes of patients with severe and steroid-resistant asthma. However, these biologicals only target effector molecules at the end of the inflammatory cascade and therefore do not have the capacity to cure the disease.

Further upstream interference in the inflammatory cascade may have the ability to not only dampen downstream effector responses but also redirect those responses towards a more tolerogenic profile. Novel therapeutics based on microbial adjuvants that target DC function, form promising candidates, as DCs determine the fate of effector versus tolerogenic T cell responses. Studies so far show that DCs are a heterogeneous group of cells, consisting of several subsets with very diverse immune-driving abilities, an activity which is very plastic and depends not only on the subset, the (mucosal) tissue location, but also on the signals they encounter in the micro-environment. Although markers so far used to distinguish different DC subsets in mice and humans were different, the functions and behavior of those DC subsets are relatively similar across species, which is helpful in determining the role of the different DC subsets through fundamental research.

A few of the initiatives applying microbial, DC-targeting adjuvants, have shown some encouraging results (Fig. 1), though proof of principle in larger patient groups seems more difficult to reach. This may again suggest that these treatments are mostly effective in subgroups of patients. However, until now, delivery systems to those DC subsets more crucially involved in the initiation of allergic responses have not been explored, nor has the choice of adjuvant been geared towards those that preferentially act on specific and relevant DC subsets. In addition, since immune tolerance to achieve prevention of allergic diseases would preferably be induced early in life, and since DCs from infants may respond slightly differently, it would be important to also explore those adjuvants that are able to modulate DC function in early life. Regarding the choice of DC-targeting adjuvants, it would be interesting to follow some of the new developments in the cancer field [154], where substantial efforts have been made to develop DC-targeting therapies. An important difference is, however, that in cancer, the goal of such therapies is to activate DC function, while in asthma DC-targeting therapy is to establish a more tolerogenic activity. Nevertheless, crossing border activities between different disease areas would help advance DC-targeting therapy for asthma and result in novel avenues for the development of DC-targeting therapies.
